# Nanoparticle-Hydrogel Composite Drug Delivery System for Potential Ocular Applications

**DOI:** 10.3390/polym13040642

**Published:** 2021-02-21

**Authors:** Xuan-Ling Hsu, Lien-Chen Wu, Jui-Yang Hsieh, Yi-You Huang

**Affiliations:** 1 Department of Biomedical Engineering, National Taiwan University, Taipei 10051, Taiwan; r07528030@ntu.edu.tw (X.-L.H.); d03548026@ntu.edu.tw (J.-Y.H.); 2 Department of Orthopaedics, School of Medicine, College of Medicine, Taipei Medical University, Taipei 11031, Taiwan; d98548019@ntu.edu.tw; 3 Department of Orthopedics, Shuang Ho Hospital, Taipei Medical University, Taipei 23561, Taiwan; 4 Department of Biomedical Engineering, National Taiwan University Hospital, Taipei 10051, Taiwan

**Keywords:** PLGA, hyaluronan, nanoparticle, injectable hydrogel, composite drug delivery system

## Abstract

Intravitreal injections are clinically established procedures in the treatment of posterior eye diseases, such as wet age-related macular degeneration (wet AMD) which requires monthly intravitreal injections of anti-vascular endothelial growth factor (anti-VEGF) protein drugs that can lead to complications due to frequent dosing. In this study, we designed a composite drug delivery system (DDS) consisting of drug-loaded poly (lactide–*co*–glycolide) (PLGA) nanoparticles and a chemically crosslinked hyaluronan hydrogel to reduce the dosing frequency. The morphology, size, composition, and drug loading efficiency of the prepared nanoparticles were characterized. The properties of the modified hyaluronan polymers used were also examined. The degree of swelling/degradation and controlled release ability of the hyaluronan hydrogel and the composite DDS were identified using bovine serum albumin (BSA) as a model drug. The results show that this system can retain 75% of its wet weight without losing its integrity and release the model drug at the rate of 0.4 μg/day for more than two months under physiological conditions. In addition, the nanoparticulate formulation of the system can further improve bioavailability of the drugs by penetrating deep into the retinal layers. In conclusion, the proposed composite DDS is easily prepared with biocompatible materials and is promising for providing the sustained release of the protein drugs as a better treatment for ocular neovascular diseases like wet AMD.

## 1. Introduction

Choroidal neovascularization is the main cause of wet age-related macular degeneration (wet AMD). Choroidal neovascularization results from an abnormally high amount of vascular endothelial growth factor (VEGF), which leads to angiogenesis of the choroid vascular, which penetrates and damages photoreceptor cells on the retina and eventually causes blindness [1,2,3]. Wet AMD is a common ocular degenerative disease in people over 50 years old [4]. It is predicted that wet AMD might affect 2.88 billion people by 2040 [5]. The current treatment for wet AMD is intravitreal injection of anti-VEGF protein drugs. The most commonly used anti-VEGF drug is bevacizumab (Avastin) as an off-label use, due to its relatively low cost. However, due to its short half-life in the vitreous humor of seven to nine days [6,7], frequent administration is needed to maintain the effective drug concentration. This regime not only leads to many complications but also causes psychological and financial burdens on patients and their families [8]. Thus, many drug delivery systems (DDS) have been developed to provide sustained and prolonged delivery of the drug to the retina [9,10,11,12]. Hydrogel can be easily injected into the vitreous chamber to treat posterior ocular diseases. However, the gelation time must be carefully modulated. For thermosensitive hydrogel, the sol-to-gel transition processes produce an expulsion of water resulting in a major loss of hydrophilic drug loaded in the polymer network, which eventually leads to a burst release. The protein drugs may be destabilized due to the temperature-dependent hydrophobic character of the system. Recently, Re-Vana Therapeutics have developed an in situ forming photo-crosslinkable gel-based system (OcuLief) and a preformed photo-crosslinked implant (EyeLief), which were proved to release anti-VEGF drugs (bevacizumab) for up to 3 months [13]. Yet, photosensitive hydrogels also require a fast gelation procedure to prevent a burst release of the drug. The high intensity light and photo-initiator used to conduct the reaction may damage the protein therapeutics, and the byproducts generated from the reaction may be toxic to the surrounding tissues [14]. Nanoporous polymeric thin films are also considered to be a good candidate to encapsulate large molecules, such as proteins [15,16,17,18,19]. However, the drying process of the films must be explored to prevent thermolabile protein drugs from degradation. Nondegradable implants including a micropump system (Posterior MicroPump, Replenish) and port delivery system (PDS, Genentech) were also developed to access controlled release of protein therapeutics [20,21]. The major concern for these systems is the surgery required for the implantation, which may induce a series of complications, such as vitreous hemorrhage and endophthalmitis.

Polymeric particles are made of biocompatible and biodegradable polymers. For example, poly (lactide–*co*–glycolide) (PLGA), which has been approved by the US Food and Drug Administration (FDA) for biomedical use, has good biocompatibility and various degradation rates. The desired degradation rate can be achieved by varying the ratio of lactic acid and glycolic acid in the PLGA polymer chains. As a result, many studies have used PLGA as a drug carrier for various types of therapy, including protein drug therapy [22,23,24]. In particulate DDS, particle size can affect the drug loading amount, drug release rate, biocompatibility, and the ability of the particles to penetrate tissues [25]. Although smaller particles have lower drug loading amounts than larger particles, they provide higher tissue penetration [25]. Particles with a diameter of <200 nm have been proved to penetrate from vitreous to retinal layers, which can further prolong the half-life of the NPs in the vitreous humor [26]. As such, nanoparticles are more effective for the long-term delivery of protein drugs to the posterior segment than microparticles, which have a shorter retention time and may cause visual disturbance from microparticle sedimentation on the retinal surface [27]. Although NPs can extend and control the release of protein drugs, they are highly dynamic in the vitreous humor, reducing biocompatibility and increasing the clearance rate of drug-laden particles. As a result, a platform is required to localize and prevent the NPs from moving freely in the posterior segment and provide the NPs with a stable deposit for longer retention [28].

Hydrogels are three-dimensional polymer networks that can absorb a large amount of aqueous solution without losing their integrity. These hydrogel networks provide a hydrophilic environment for the stable storage of protein drugs, cells, and nanoparticles by preventing them from enzymatical degradation and hydrophobic interaction, which may harm the cargos [28,29]. In addition, the porous structure of the hydrogels supports a high payload of the contents [25]. Hydrogels can be made of natural polymers [30,31], synthetic polymers [32,33], or a combination of the two [11]. Natural polymers tend to be more degradable than synthetic ones since they have more hydrolysable linkages and can be subjected to enzymatic degradation within a living body [25]. For drug delivery to the posterior segment of the eye, injectable hydrogels are preferred due to the minimally invasive method required to transport them to the vitreous humor. Injectability can be achieved utilizing thermosensitive gels, photosensitive gels, micro/nanogels, and in situ forming hydrogels [12]. In situ forming hydrogels are formed by physically crosslinking through noncovalent interactions or by chemically coupling reactive functional groups on the polymer chains [12,29,34]. Chemically crosslinked gels are favored for long-lasting DDS since they have a slower degradation rate than physically crosslinked gels. Recently, click reactions were widely used for preparing in situ forming chemically crosslinked gels include the Diels-Alder reaction, copper(I)-catalyzed alkyne-azide reaction, and thiol-ene reaction [35]. Among these reactions, the thiol-ene reaction is most attractive for in situ gel forming, since it is simpler for generating thiols and alkenes on the polymer chains. Furthermore, the thiol-ene click reaction requires only mild conditions and needs no catalysts to proceed, forming covalent bonds between polymer chains without producing byproducts [29]. Despite these advantages of in situ forming hydrogel, a fast release rate is observed when encapsulating protein drugs directly into the hydrogel. This results from the gels’ inherently high water content, so hydrophilic protein drugs are easily diffused to the surroundings as the hydrogel networks swell [36]. Therefore, it is necessary to slow the diffusion rate of loaded protein drugs to extend the release profile of the DDS.

The disadvantages of individual drug delivery systems include high initial burst release, toxicity induced by particle migration, and rapid diffusion of hydrophilic protein drugs from a swelled hydrogel. In recent years, the combination of polymeric particles and injectable hydrogels has been broadly studied to address these issues. By integrating these DDSs, hydrogels are a secondary barrier that can reduce the initial burst release observed in a polymeric particle system, and also provide a stable deposit for localizing the particles, which can protect particles from clearance and degradation [37]. In addition, the tunable drug loading amount and degradation rate of the polymeric particle system further improve the overall therapeutic effect of the composite DDS [36]. The combination of the DDSs not only diminishes the drawbacks of each DDS but also creates some additional properties, not seen in the individual systems [38,39,40]. Recently, a MPEG-PCL micelles/F-127 hydrogel [41] and chitosan nanoparticles/GelMA photon-crosslinked hydrogel [42] were proposed to improve the delivery of drugs from topical ocular formulations and proangiogenesis growth factor containing scaffold, respectively. Studies of PEO-PLA nanoparticles/HA-MC hydrogel [43], PLGA microparticles/HA hydrogel [44], and PCL nanoparticles/Poloxamer 407 thermosensitive hydrogel [45] were also proposed to deliver drugs into the aqueous humor of the eyes, knee joint, and skin. However, drug delivery targeted to the posterior segment of the eyes is still unclear. Kang-Mieler et al. developed a DDS composed of PLGA microspheres and crosslinked N-isopropylacrylamide (NIPAAm)-based thermosensitive hydrogel capable of extending the retention time of anti-VEGF drugs in rabbit eyes for up to 200 days [36,37,39,46]. However, the NIPAAm monomer used was considered to be retinal toxic and nondegradable [47,48,49]. Hyaluronan (HA) can be enzymatically degraded by the living body. As a major component of the vitreous humor (100~400 µg/mL), HA is widely used in ophthalmology as a vitreous substitute [48,50]. Hyaluronan can be chemically modified through the carboxylic groups, hydroxyl groups, and N-acetyl groups [51]. Moreover, hyaluronan-based hydrogel can be easily prepared with modified hyaluronan and either natural or synthetic polymers [51], forming chemically crosslinked hydrogel via click reaction including Schiff base reaction [52,53], thiol-ene reaction [54,55], and Diels-Alder reaction [56]. In recent years, HA hydrogel has been widely used in the development of drug delivery systems [11,31,32,54], tissue engineering scaffold [52,53,55,56], and the regenerative cell matrix [54,55].

This study develops a biodegradable composite DDS consisting of PLGA nanoparticles and injectable HA hydrogel for long-term delivery of protein drugs to the posterior segment of the eye. The physical and chemical properties of this composite DDS are characterized. Biocompatibility of the system is investigated by coculturing the extracts of the DDS with human retinal pigmented epithelial cells (ARPE-19). Finally, the feasibility of the sustained and prolonged release of the proposed DDS is demonstrated using bovine serum albumin (BSA) as a model drug in vitro.

## 2. Materials and Methods 

### 2.1. Materials

Bovine serum albumin (BSA), PLGA (LA:GA = 75:25, *M*_W_ = 66,000–107,000), Tween-80, dichloromethane (DCM), ethyl acetate (EA), Pluronic F-68 (*M*n = 8,400), D-mannitol, divinyl sulfone (DVS), and DL-dithiothreitol (DTT) were purchased from Sigma-Aldrich (St. Louis, MO, USA). Sodium hyaluronate (HA) (*M*_W_ = 100,000) was obtained from Seedchem (Camberwell, Australia). ARPE-19 cells were purchased from ATCC (Manassas, VA, USA). Fluoresbrite yellow green nano/microspheres with diameters of 50 nm and 2 µm were purchased from Polysciences (Warrington, PA, USA) for studying the release behavior in the hydrogel. All the chemical reagents in the experiments were used without further purification.

### 2.2. Preparation of Bovine Serum Albumin Loaded PLGA Nanoparticles

Bovine serum albumin (BSA) was encapsulated in PLGA nanoparticles using a modified double-emulsion solvent-evaporation method, according to previous research [23,24]. In brief, 400 µL of 2.5% *w*/*v* BSA solution in DI water containing 5% *w*/*v* Tween-80 was emulsified into 4 mL of 5% *w*/*v* PLGA solution dissolved in an organic solvent mixture of DCM and EA (ratio of 1 to 3) using a probe sonicator (9 W, 45 s, XL-2000 series, MISONIX). The water-in-oil emulsion was then added dropwise into 8 mL of 1% *w*/*v* Pluronic F-68 solution and sonicated at 9 W for another 90 s. The double emulsion was stirred overnight at room temperature to allow evaporation of the organic solvent and hardening of the NPs. To collect the final NPs, the solution was centrifugated (22,000 g, 4 °C, and 15 mins) and washed three times with DI water. Finally, the BSA-laden PLGA NP solution was mixed with 0.75 mL of 4% mannitol solution and freeze-dried.

### 2.3. Modification of Hyaluronan Polymer

Divinyl sulfone functionalized HA (HA-VS) and thiolated HA (HA-SH) polymer were prepared according to previous research [34,57]. Briefly, HA-VS was made by reacting 2% *w*/*v* HA with 1.25 times molar amount of DVS at 0.1 M NaOH for 2 mins 45 secs and stopped by adding 1 M HCl to bring the system pH to 5. The solution was dialyzed against DI water (~pH 5.3) for 4 days using a dialysis bag (MWCO 3500, Cellu Sep, MFPI, Seguin, TX, USA). The purified HA-VS was further sterilized by filtrating through a 0.22 µm syringe filter, freeze-dried, and stored at −20 °C before use. H^1^-NMR spectrum of the HA-VS polymer was determined on a 500 MHz AVIII-500 NMR Spectrometer (Bruker, German) by dissolving the sample in D_2_O. The degree of modification (DM) of the HA-VS polymer indicates the number of vinyl sulfone groups of each HA unit. The DM was determined by comparing the integrated area under curve at the peak of the vinyl sulfone carbon−carbon double bond (6.8 ppm) to the area under the peak of N-acetyl glucosamine proton of native HA (1.9 ppm).

To prepare HA-SH polymer, an adequate amount of phosphate buffer (0.1 M, pH 7.4) was added into the purified HA-VS solution to bring the system pH to 7.4. The solution was then reacted with 10 times the molar amount of DDT dissolved in 0.1 M phosphate buffer (70% *w*/*v*) for 20 mins in N2. The HA-SH solution was purified using a dialysis bag (MWCO 3500, Cellu Sep, MFPI) against DI water for 4 days and further sterilized by filtrating through a 0.22 µm syringe filter, freeze-dried, and stored at −20 °C before use.

### 2.4. Preparation of the Composite Drug Delivery System

The HA-VS polymer was dissolved in PBS containing a calculated amount of BSA-laden PLGA NPs. The HA-SH polymer was dissolved in PBS at the same volume of HA-VS polymer solution. The composite DDS was formed by blending these two precursor solutions at room temperature. Table 1 indicates the abbreviations of the tested groups and their compositions. Twenty milligrams of the BSA-laden NPs contained about one milligram of BSA according to the loading efficiency calculated in the following sections. As such, groups A-BSA, A-NP, B-BSA, and B-NP were loaded with the equivalent amount of the model drug, which was 1 mg of BSA.

### 2.5. BSA-Laden PLGA NP Characterization

The morphology of BSA-laden PLGA NPs was increased by scanning electron microscope (S-4800, Hitachi, Japan). The size of the NPs was calculated from SEM images using Image J (NIH). The hydrodynamic diameter of the NPs was examined by ZetaSizer (Nano ZS, Malvern Panalytical, UK). Compositions of the BSA-laden PLGA NPs were confirmed by Fourier transform infrared spectrometer in attenuated total reflectance model (Nicolet Summit PRO, Thermofisher, Waltham, MA, USA) at a resolution of 0.48 cm^−1^ in the frequency interval of 400 to 4000 cm^−1^.

The amount of BSA entrapped in the PLGA NPs was calculated as follows. In brief, after centrifugation of the NPs, the supernatants were collected, diluted if necessary, and detected using bicinchoninic acid assay (Biosciences, USA) performed according to the product manual. BSA entrapped in the NPs equaled the total amount of BSA added minus the amount of BSA in the supernatants. The entrapment efficiency (EE) was calculated as follows:

(1) $$\mathrm{Entrapment}\;\mathrm{efficiency}\;(\%)=\frac{\mathrm{BSA}\;\mathrm{added}\;(\mathrm{mg})-\mathrm{BSA}\;\mathrm{in}\;\mathrm{the}\;\mathrm{supernatant}\;(\mathrm{mg})}{\mathrm{BSA}\;\mathrm{added}\;(\mathrm{mg})}\times 100\%.$$

Loading efficiency (LE) was calculated as follows:

(2) $$\mathrm{loading}\;\mathrm{efficiency}\;(\%)=\frac{\mathrm{BSA}\;\mathrm{added}\;(\mathrm{mg})-\mathrm{BSA}\;\mathrm{in}\;\mathrm{the}\;\mathrm{supernatant}\;(\mathrm{mg})}{\mathrm{Total}\;\mathrm{amount}\;\mathrm{of}\;\mathrm{the}\;\mathrm{BSA}\;\mathrm{loaded}\;\mathrm{PLGA}\;\mathrm{NPs}\;(\mathrm{mg})}\times 100\%.$$

### 2.6. HA Hydrogel Gelation Time and Injectability Assessment

The gelation time of various hydrogel compositions was increased according to the tube inversion test [58]. As Table 1 shows, six different groups with different initial polymer concentrations and different contents were tested. A total volume of 200 µL of the precursor solutions was prepared for each group, separately. HA-VS and HA-SH polymer solutions were blended thoroughly and placed at room temperature for gelation. At one min intervals, the tube containing the blended polymer solution was inverted for 30 secs, if no flow of the solution was observed, it was affirmed that the solution had transformed into its gel state. All groups were performed in triplicate. To demonstrate injectability of the hydrogels, group A and group B hydrogels were prepared and loaded to 1 mL syringe with needles of 260 µm (26 gauge) and 108 µm (32 gauge) inner diameter. After gelation, the hydrogels were pushed out of the syringe and photographed.

### 2.7. In Vitro Swelling and Degradation Properties

A total volume of 200 µL of hydrogel was prepared and weighed (*W*_0_) for each group. The prepared hydrogels were immersed in 2 mL PBS and incubated at 37 °C. At specific time points, the surface PBS was removed carefully and the hydrogel was weighed (*W*_t_). The degree of swelling and degradation were calculated as follows:

(3) $$\mathrm{Degree}\;\mathrm{of}\;\mathrm{swelling}\;(\%\;w/w)\;\mathrm{and}\;\mathrm{degradation}\;(\%\;w/w)=\frac{W_{t}-W_{0}}{W_{0}}\times 100\%.$$

Positive values and negative values were considered to be the degree of swelling (% *w*/*w*) and the degree of degradation (% *w*/*w*), respectively. All groups were performed in duplicate.

### 2.8. Cytotoxicity

Cytotoxicity of the composite DDS was elevated by MTT assay using human retinal pigmented epithelial (ARPE-19) cells. ARPE-19 cells were seeded in a 96-well plate at a density of 10,000 cells/well for all experiments. Cytotoxicity was determined by the extract exposure method [59]. Total volumes of 200 µL for the tested groups in Table 1 were prepared separately, and an additional group of group NP containing 20 mg of BSA-laden PLGA NPs dissolved in 200 µL PBS was also prepared. All groups were immersed in 400 µL fresh PBS and incubated at 37 °C. At specific time points, 10 µL of the extracts were added to ARPE-19 culture wells containing 90 µL of the fresh media for 48 hrs. Since the volume ratio of human vitreous humor to the fresh PBS used to immerse the tested groups was 10, the extracts were performed with a 10-fold dilution when added into the ARPE-19 cell culture. After 48 hrs of incubation, extracts were removed and the cells were washed by fresh PBS three times. Subsequently, 100 µL of 0.5% *w*/*v* MTT reagent was added to the wells and incubated for 4 hrs at 37 °C. After 4 hrs, the MTT reagent was removed, 100 µL of DMSO was added, and the plate was shaken for 5 mins before further detection. Absorbance of all wells was recorded at 540 nm using a microplate reader, cells cultured with only fresh media were used as control groups. All groups were performed in triplicate. Cell viability of each group was calculated as follows:

(4) $$\mathrm{cell}\;\mathrm{viability}\;(\%)=\frac{{\mathrm{OD}}_{tested group}-{\mathrm{OD}}_{\mathrm{blank}}}{{\mathrm{OD}}_{\mathrm{ctrl}}-{\mathrm{OD}}_{\mathrm{blank}}}\times 100\%.$$

### 2.9. In Vitro Release of BSA

A total volume of 200 µL of group A-NP and B-NP hydrogels were prepared separately. The tested groups were immersed in 2 mL of fresh PBS and incubated at 37 °C. At specific time points, 1 mL of PBS buffer was removed and 1 mL of fresh PBS was added. To detect the released amount of BSA, bicinchoninic acid assay (Biosciences, USA) was conducted according to the product manual. All groups were performed in triplicate.

### 2.10. Micro/Nanoparticles Behavior in the Hydrogel

To elevate the distribution of the microsized and nanosized particles in the hydrogel, diameters of 50 nm and 2 µm for the fluorescent polystyrene particles were used in the following experiments. A total volume of 5 µL of the tested groups listed in Table 2 were prepared separately, as described in Section 2.4. The prepared hydrogels were allowed to gel at room temperature, and after gelation, 100 µL of fresh PBS was added and the tested groups were incubated at 37 °C. At specific time points, the gels were observed under a fluorescent microscope (IX70, Olympus, Japan) and photographed.

For group A-NP-50nm and group B-NP-50nm, the media were observed separately at a constant exposure time of 1 sec for all groups at different time points. To quantify the released amount of PS fluorescent nanoparticles, the mean gray value of each photograph (media only) was increased using Image J (NIH) in the same picture area.

### 2.11. Statistical Analysis

All data presented in the study are indicated as mean ± SD. Statistical analysis was performed with paired samples t-test. *P* values <0.05 are considered statistically significant.

## 3. Results and Discussion

### 3.1. Characterization of BSA-Laden PLGA Nanoparticles

**Figure 1:**
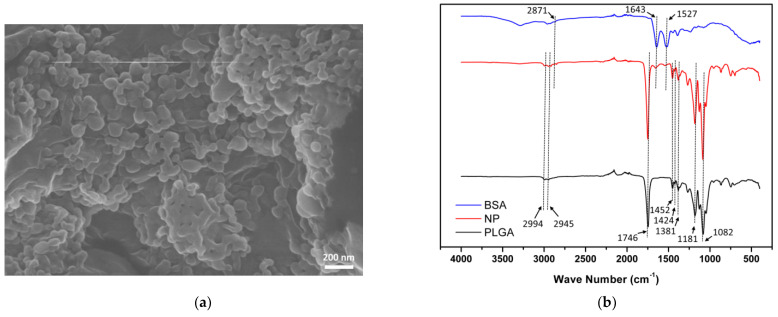
Characterization of BSA-laden PLGA nanoparticles. (**a**) SEM image of BSA-laden PLGA NPs. The nanoparticles were spherical with a smooth surface, yet some aggregation occurred due to freeze-drying procedure. (**b**) ATR-FTIR spectra of bovine serum albumin (BSA), BSA-laden PLGA NPs (NP), and PLGA. PLGA spectra (black curve) had three characteristic peaks attributed to the three different monomer sequences of PLGA polymer chains of lactide−glycolide at 1381 cm^−1^, glycolide-glycolide at 1424 cm^−1^, and lactide-lactide at 1452 cm^−1^. BSA spectra (blue curve) had typical protein characteristic peaks at 1643 cm^−1^ (amide I) and 1527 cm^−1^ (amide II). NP spectra (red curve) had the characteristic peaks of both BSA spectra and PLGA spectra, indicating successful encapsulation of BSA into PLGA nanoparticles.

The morphology of BSA-laden PLGA nanoparticles was examined by SEM. According to the SEM image (Figure 1a), BSA-laden NPs were spherical with a smooth surface, yet some aggregation occurred, probably due to the freeze-drying procedure. The size of BSA-laden NPs measured by Image J was 54.81 ± 7.95 nm, and the hydrodynamic diameter of NPs was 184.97 ± 29.54 nm examined by ZetaSizer. Encapsulation efficiency (EE) and loading efficiency (LE) of the BSA-laden NPs were 20.53 ± 9.72 % and 2.14 ± 2.12 %, respectively.

Composition of as-prepared BSA-laden NPs was confirmed by ATR-FTIR spectrometry. As shown in Figure 1b, the black curve represented PLGA, which had three characteristic peaks attributed to the three different monomer sequences of PLGA polymer chains of lactide-glycolide at 1381 cm^−1^, glycolide-glycolide at 1424 cm^−1^, and lactide-lactide at 1452 cm^−1^. PLGA spectra also had several characteristic peaks corresponding to specific chemical structures in the PLGA polymer including 1082 cm^−1^ and 1181 cm^−1^ (C–O bond), 2945 cm^−1^, and 2994 cm^−1^ (CH bond), and 1746 cm^−1^ (C=O bond). The blue curve indicated BSA, which had the typical protein characteristic peaks at 1643 cm^−1^ (amide I) and 1527 cm^−1^ (amide II), and a visible peak at 2871 cm^−1^ was attributed to the CH bond. The red curve indicated BSA-laden PLGA NPs, which had the characteristic peaks of both BSA and PLGA polymers, indicating successful encapsulation of BSA into PLGA nanoparticles.

### 3.2. Chemical Structure of the Modified HA Polymer

**Figure 2:**
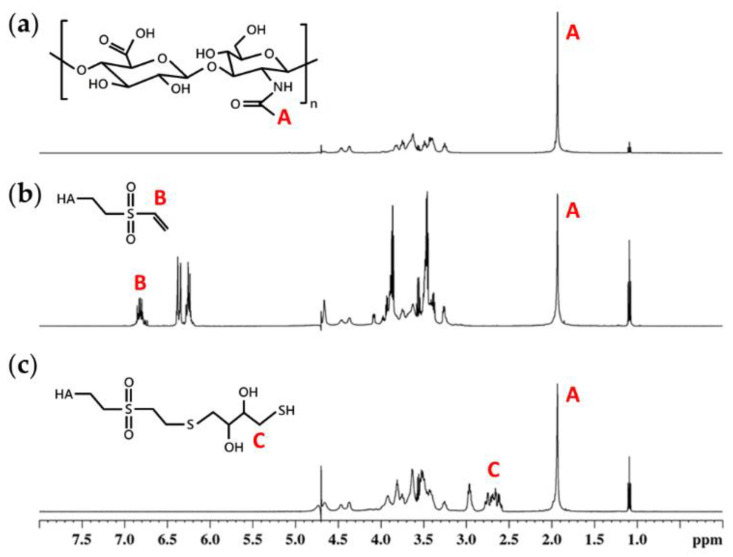
H^1^-NRM spectra of (**a**) hyaluronan (HA), (**b**) divinyl sulfone functionalized HA (HA-VS) polymer, and (**c**) thiolated HA (HA-SH) polymer. The degree of modification of HA-VS polymer was 29 % calculated by comparing the area under peak at 6.8 ppm (carbon−carbon double bond of vinyl sulfone groups), indicated by B, to peak at 1.9 ppm (*N*-acetyl glucosamine of HA), indicated by A.

H^1^-NMR was performed to confirm the chemical structure of the modified HA polymers (Figure 2). Each H^1^-NMR spectrum had a characteristic peak at 1.9 ppm indicating the N-acetyl glucosamine proton of native HA. Peaks at 6.2, 6.4, and 6.8 ppm were attributed to the carbon−carbon double bond of vinyl sulfone groups (VS), indicating the success of grafting vinyl sulfone groups to HA chains. The degree of modification (DM) was calculated by comparing the integrated area under curve at 6.8 ppm and 1.9 ppm, which was 29% for HA-VS polymer. For HA-SH polymer, thiol groups (SH) had several unique peaks around 2.5 to 3.0 ppm. These peaks could not be used to calculate the DM of HA-SH polymer since thiol groups are easily oxidized under atmospheric conditions. However, the disappearance of peaks at 6.2, 6.4, and 6.8 ppm indicated that all of the vinyl sulfone groups were grafted with thiol groups. Since HA-SH polymer was synthesized from HA-VS polymer, the degree of modification of HA-SH polymer was 29%, the same as HA-VS polymer.

### 3.3. In Situ Chemically Crosslinked HA Hydrogel Formation

**Figure 3:**
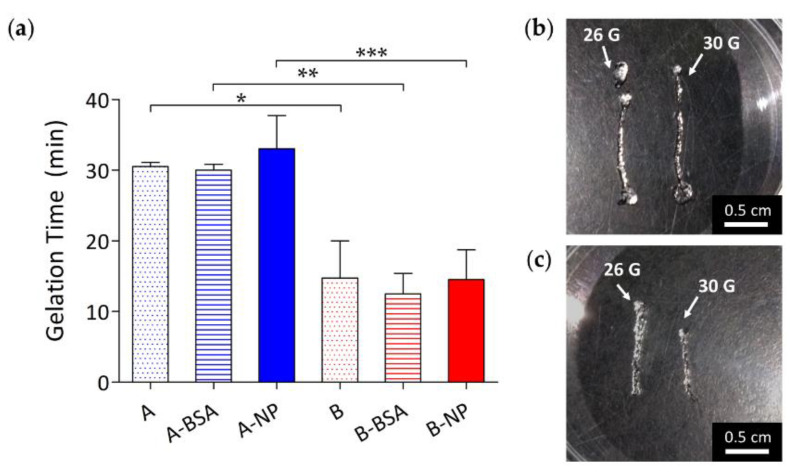
(**a**) Gelation time of hydrogels with polymer concentrations of 1.5% (A groups) and 3% (B groups) loaded with nothing (A and B), BSA (A-BSA and B-BSA), and BSA-laden PLGA NPs (A-NP and B-NP). Gelation times of the A groups were significantly longer than the B groups due to the higher initial polymer concentration of the B groups. Injection feasibility of group A and group B hydrogels are shown as (**b**) and (**c**), respectively. These hydrogels can be easily injected through 26 G and 30 G needle. (*: *p* ≤ 0.05; **: *p* ≤ 0.01; ***: *p* ≤ 0.001).

The gelation time of various HA hydrogels was determined by tube inversion test [58]. Gelation time of in situ chemically crosslinked HA hydrogel was mainly dependent on the polymer concentration. As shown in Figure 3a, hydrogels with lower polymer concentration had a longer gelation time of about 30 mins. The gelation times of groups A, A-BSA, and A-NP were 30.50 ± 0.58 mins, 30.00 ± 0.82 mins, and 33.00 ± 4.69 mins, respectively. For the hydrogels with higher polymer concentration, gelation time was significantly shorter than those with lower polymer concentration (*p* < 0.05), taking about 15 mins for complete gel formation. The precise gelation times of groups B, B-BSA, and B-NP were 14.75 ± 5.25 mins, 12.50 ± 2.89 mins, and 14.50 ± 4.20 mins, respectively. This phenomenon contributed to the higher number of functional groups in the B groups, resulting from the higher initial polymer concentration of the B groups. A rapid gelation time is preferred for encapsulating protein drugs in situ to prevent the drugs from diffusing into surrounding areas [12,60]. However, from the clinical point of view, a longer gelation time is more convenient for the operator to carry out the procedure without the precursor solution becoming gel, which is much more difficult to push through a needle for intravitreal injection.

### 3.4. In Vitro Swelling/Degradation Properties of Chemically Crosslinked HA Hydrogel

**Figure 4:**
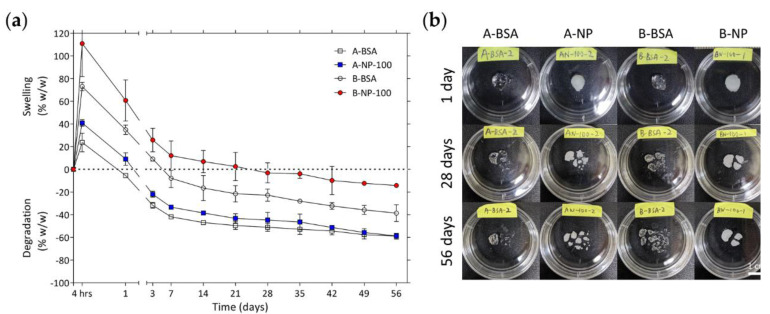
(**a**) Degree of swelling/degradation for various groups loaded with BSA and BSA-laden PLGA NPs. (**b**) Shows enlarged views of each groups after 1 day, 28 days, and 56 days incubation in PBS at 37 °C.

In vitro swelling and degradation properties of the gels were examined at 37 °C, and the degree of swelling and degradation were calculated by comparing the gel wet weight to its original wet weight. If the gel wet weight was heavier than its original weight, the gel was considered at its swelling state. If the gel wet weight was lighter than its original weight, the gel was considered at its degradation state. As shown in Figure 4, there was swelling in all groups within the first 4 hrs, and the B groups had more swelling than the A groups. The A groups started to degrade after 1 day and the B groups after 7 days. After 14 days, the A groups lost almost half of their original wet weight while the B groups lost only approximately 30% of their original wet weight. The faster degradation rate of the A groups might be a result of their lower crosslinking density. After 14 days, all groups underwent a slower degradation rate throughout the experiment. After 56 days of incubation, the weight losses of A-BSA, A-NP, B-BSA, and B-NP were −58.93 ± 2.51 %, −58.72 ± 0.72 %, −38.60 ± 7.45 %, and −14.27 ± 0.98 %, respectively. As noted above, the B groups initially had more swelling than the A groups, perhaps because they had higher crosslinking density and more hydrophilic hydroxyl groups than the A groups, which allowed the hydrogel to absorb more water molecules without losing its integrity, resulting in more swelling [61]. Furthermore, due to their higher polymer concentration, the B groups had more uncrosslinked polymer chains in the hydrogel networks, which led to a faster degradation rate in the first 7 days. This phenomenon disappeared when most of the uncrosslinked polymer chains degraded into the surroundings, followed by a considerably slower degradation rate after 14 days. In conclusion, the degree of swelling and degradation were mainly influenced by the crosslinking density of the hydrogels, and not affected by the content of the hydrogels.

### 3.5. Cytotoxicity of the Composite DDS

**Figure 5:**
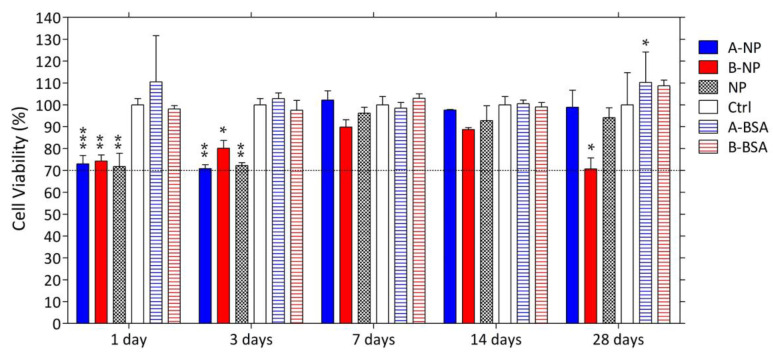
Cytotoxicity of the composite DDS to the ARPE-19 cells. The dashed line indicates 70% of cell viability. (*: *p* ≤ 0.05; **: *p* ≤ 0.01; ***: *p* ≤ 0.001).

The cell viability of ARPE-19 (human retinal pigmented epithelial) cells cultured with extracts of BSA-laden PLGA nanoparticles, chemically crosslinked HA hydrogels, and the composite DDS were also tested. Extracts of each group were collected over one month. As shown in Figure 5, extracts of blank HA hydrogels had no influence on cell viability over one month. On the contrary, extracts of groups containing BSA-laden PLGA nanoparticles (group ANP, group BNP, and group NP) had significant toxicity to ARPE-19 cells at one day and three days, but this effect was not observed at one week and two weeks. However, group BNP showed a significantly higher toxicity to ARPE-19 cells than the control group after one month. This may be because the organic solvent used to dissolve PLGA in the synthesis process was still present in the PLGA nanoparticles, so as incubation time increased, the residual organic solvent diffused into the surroundings, making the extracts of these groups toxic to the ARPE-19 cells. This toxicity could be reduced by increasing the evaporation time for organic solvent during synthesis or by putting the PLGA nanoparticles into a drying cabinet for sufficient time before use.

For group BNP, the toxicity reemerging at one month might also contribute to the toxicity of the residual free vinyl sulfone groups [41,42]. Group BNP tended to be more toxic than group ANP and B. That group BNP had a higher initial HA-VS polymer concentration and more BSA-laden NPs might restrict the crosslinking process, increasing un-reacted vinyl sulfone groups compared to groups ANP and B. Vinyl sulfone groups are electrophiles that can bind or alkylate proteins which are crucial to cellular functions, and therefore lead to decreased cell viability. Despite the susceptibility to toxicity of uncrosslinked vinyl sulfone groups, further study must be conducted to measure the amount of free vinyl sulfone groups in the hydrogel in order to confirm whether the concentration of residual vinyl sulfone groups is at a toxic level. To reduce toxicity, the ratio of thiol groups to vinyl sulfone groups can be increased to ensure that most of the vinyl sulfone groups are crosslinked with thiol groups. Finally, although some cytotoxicity was observed, the cell viability calculated was still higher than 70%, as specified by ISO 10993-5, for all tested groups, indicating that these groups were still non-cytotoxic to ARPE-19 cells.

### 3.6. In Vitro Release Study

**Figure 6:**
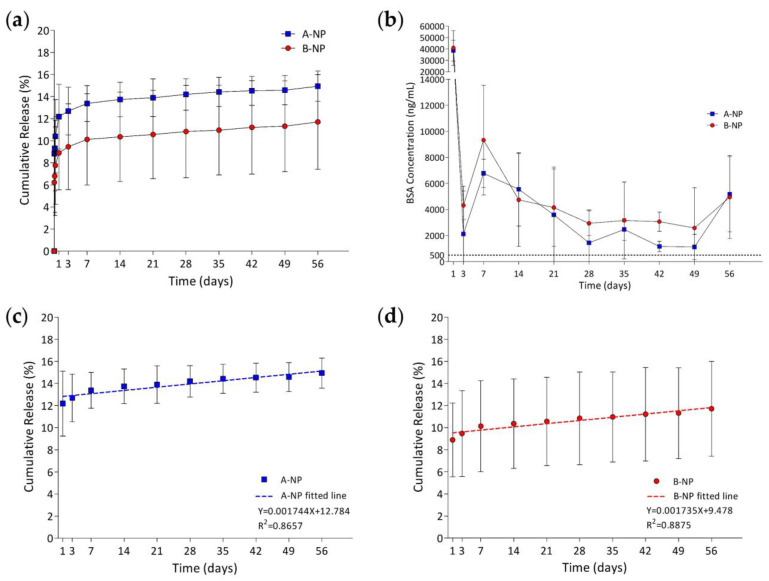
(**a**) Cumulative release percentage and (**b**) BSA release concentration of group A-NP and group B-NP. The dashed line indicates the minimum anti-VEGF drugs needed to block VEGF effectively, which was 500 ng/mL. Linearly fitted lines of group ANP and group BNP releasing BSA are shown in (**(c)**,**d**), respectively.

In vitro bovine serum albumin (BSA) released from the composite DDS was studied in PBS at 37 °C. Figure 6 shows the in vitro release profile of composite DDS with different polymer concentrations present in percent and concentration. The drug-loaded amount was 959.82 ± 28.41 µg and 978.37 ± 13.46 µg for group A-NP and group B-NP, respectively. For both groups, BSA was rapidly released within the first 24 hrs, followed by a steady linear release until the end of the study (56 days). At the initial burst (IB) release state, the IB release percent was 13.74 ± 1.63 % and 7.48 ± 3.20 % of the loaded drugs for group A-NP and group B-NP, respectively. No significant differences were found between the two groups (*p* < 0.05). After one day, the release profile entered a steady release state with a release rate 0.419 µg/day and 0.416 µg/day calculated by linear regression analysis for group A-NP and group B-NP, respectively (Figure 6c,d). According to previous research [62], both groups can maintain a concentration higher than the lowest therapeutic concentration required for blocking VEGF. At the end of the study, the final drug release percent was 14.94 ± 1.36 % and 11.70 ± 4.29 % of the loaded drugs for group A-NP and group B-NP, respectively.

Initial burst release was caused by the increased mesh size as the hydrogel swelled. The increased mesh size allowed the drugs on the surface of the nanoparticles and the free drugs in the hydrogel network to diffuse into the surroundings. Group B-NP had a lower IB release amount, probably due to the denser hydrogel network that restrained drugs from diffusing, even the hydrogel had swelled bigger than group A-NP. After the initial burst release, a zero-order release was achieved as PLGA nanoparticles degraded for both groups for two months. It is noteworthy that the experiment was terminated at 56 days due to the BSA concentration detection limit, however, according to the slope of the release curves calculated by linear regression analysis, a longer release time can be expected. The biphasic release profile presented by the composite DDS might be beneficial for clinical use of anti-VEGF therapy for AMD. Since VEGF is critical in choroidal vasculature, continuous exposure to a high concentration of anti-VEGF antibody might have some unfavorable effects on the surrounding choroidal tissues [33,63]. Thus, the biphasic release mechanism is preferred since the IB release state can block the initial higher amount of VEGF and keep anti-VEGF drugs at a concentration that is lower but sufficient for continuous VEGF blockage in the second steady release state to prevent surrounding tissues from toxic drug concentration. The final drug release percent was below 20% for both groups, which might be due to the method used to determine the drug encapsulation efficiency by assuming that all of the PLGA polymer added had transformed into the final nanoparticles collected. Therefore, the calculated encapsulation efficiency was higher than the real EE. Moreover, there must be further loss of BSA-laden nanoparticles during the loading of nanoparticles into the hydrogels since some of the NPs might stick to the tube surface instead of being tracked in the final composite DDS. As a result, the calculated drug-loaded amount might be higher than the real drug-loaded amount, which led to the lower final drug release percentage observed. This limitation could be overcome by dissolving BSA-laden PLGA nanoparticles in organic solvent (like DCM), to assess the precise encapsulation efficiency of the nanoparticles.

During the development of the protein delivery system, it was important to investigate the stability and activity of the released protein, since the procedure used to encapsulate drugs into nanoparticles might affect the protein integrity resulting in a loss of protein activity. Mechanisms including deamination and peptide bond hydrolysis may occur in a degrading PLGA system [64]. In previous studies, the stability and activity of anti-VEGF protein drugs still remained after encapsulation in PLGA nanoparticles [23,65]. The activity of the released proteins can be further examined by using enzyme-linked immunosorbent assay (ELISA), size exclusion chromatography (SEC), and circular dichroism (CD), for the assessment of protein activity, stability, and conformational stability, respectively [33,66]. Although there are some obvious differences between bevacizumab and the model drugs BSA, a sustained and extended release ability presented by the composite DDS can be expected. In addition, the molecular weight is higher for bevacizumab than BSA, which causes bevacizumab to be larger than BSA, so the release rate for bevacizumab must be slower than BSA increasing the total release time. In future studies, a higher initial bevacizumab-loaded amount and a higher encapsulation efficiency of nanoparticles should be conducted to enhance and prolong the release profile. The examination of the in vivo efficacy of the composite DDS in an appropriate animal model should be carried out, to further confirm the capability of the composite DDS for AMD treatment.

### 3.7. Particle Behavior in the Composite DDS

To understand the behavior of nano/microsized particles in the crosslinked HA hydrogel, fluorescent particles were loaded into the HA hydrogels with different initial polymer concentrations and the composite DDS was incubated in PBS at 37 °C. Polystyrene fluorescent particles with diameters of 50 nm and 2 µm were used. The released PS fluorescent nanosized particles from the hydrogel were difficult to distinguish from each other due to the resolution limit of the fluorescent microscope. Therefore, groups loaded with nanosized PS particles were further analyzed to obtain the intensity of green fluorescent light emitted from the PBS solution, which could then quantify the amount of PS NPs released from the gel.

**Figure 7:**
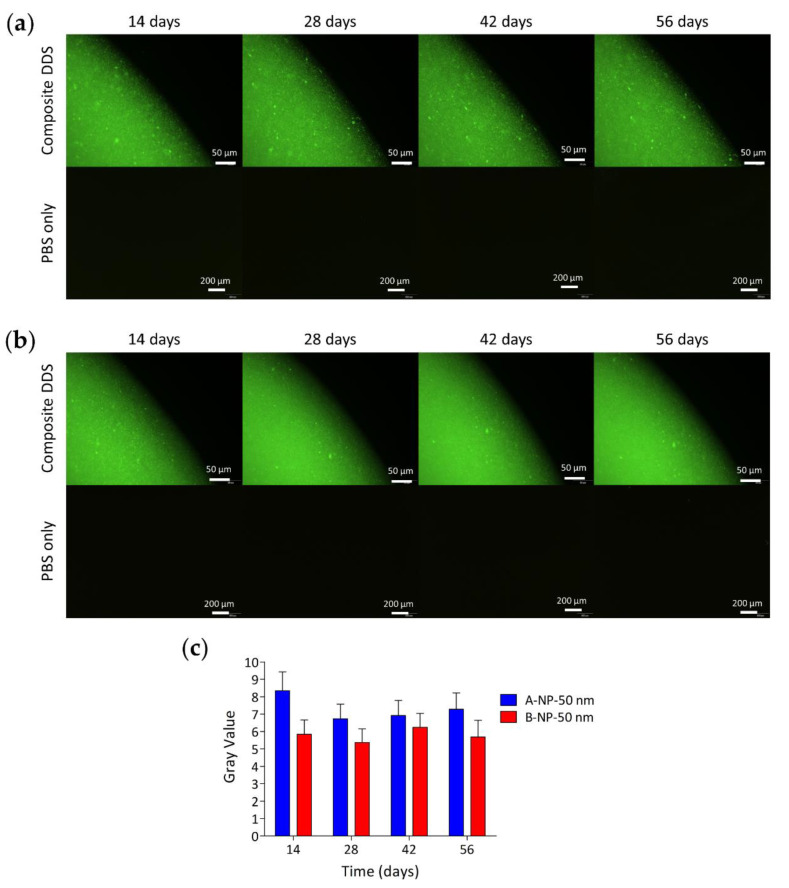
Distribution of 50 nm PS fluorescent nanoparticles in the hydrogel with a polymer concentration of 1.5% and 3% are shown in (**a**) and (**b**), respectively. (**c**) Represents the intensity of emitted fluorescent green light from the PBS solution of two hydrogel groups through 56 days of observation. The data show that PS NPs were more easily diffused to the surroundings when carried by groups with lower hydrogel polymer concentration.

**Figure 8:**
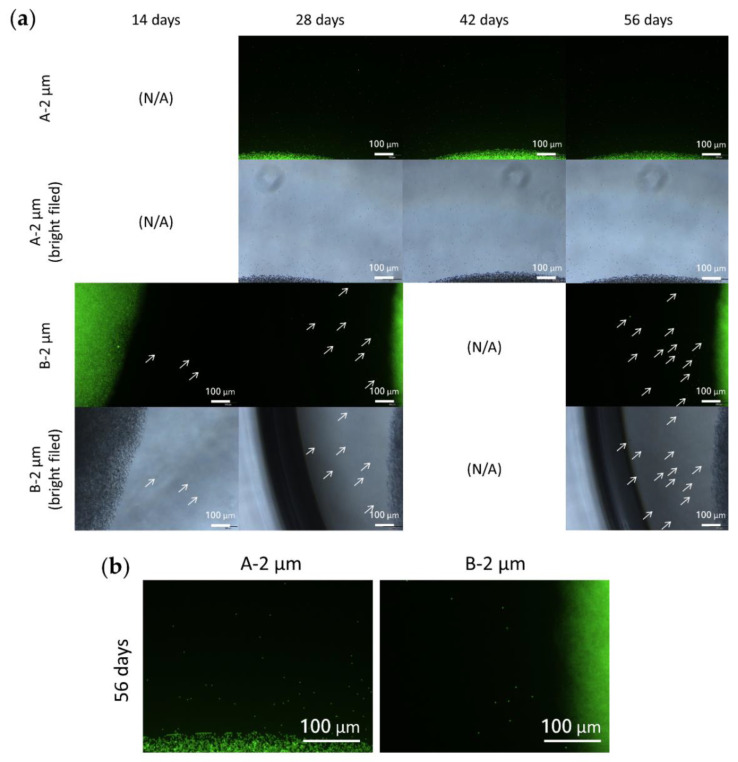
(**a**) Distribution of 2 µm PS fluorescent microparticles in the hydrogel with polymer concentrations of 1.5% and 3%. White arrows indicate the microparticles released from the hydrogel. (**b**) Enlargements of the two groups after 56 days incubation, clearly showing that PS MPs were more easily released from groups with lower hydrogel polymer concentration. This finding corresponded to the groups loaded with PS NPs.

As Figure 7 and Figure 8 show, the particle diffusion rate was slower for the B groups than for the A groups, which might have contributed to the reduced hydrogel pore size due to the higher initial polymer concentration of the B groups [67]. On the other hand, since NPs had smaller particles, they diffused more easily into the surroundings than MPs. Sakurai et al. also used PS NPs/MPs to evaluate the behavior of the particles with a diameter of 50 nm and 2 µm in rabbit eyes [27]. The elimination half-life from the posterior segment of the rabbit eyes was 5.4 ± 0.8 days and 10.1 ± 1.8 days for particles of 2 µm and 50 nm, respectively. In addition, the microsized particles (2 µm) accumulated on the retina caused turbidity in the vitreous humor one month after intravitreal injection. On the contrary, nanosized particles could still be observed in the retina tissues with clearer vitreous humor. Although there are some non-negligible differences between nondegradable PS particles and degradable PLGA particles, it can be concluded that nanosized particles are better for the long-term release of anti-VEGF drugs to the retina since they can penetrate into the retinal tissues, rather than remaining on the retina surface.

## 4. Conclusions

In this study, a composite DDS was easily prepared by combining PLGA nanoparticles with crosslinked HA hydrogel. The prepared chemically crosslinked hydrogel could gel at physiological conditions simply through blending the precursor solutions. No significant cytotoxicity was observed when the extracts of the system were cultured with ARPE-19 cells. The composite DDS with higher hydrogel polymer concentration of 3% w/v can maintain 75% of its wet weight without losing its integrity for two months, which is critical for a prolonged drug retention time. The gelation time of the composite DDS was about 15 mins, which is beneficial for clinal use since the operator can perform the injection procedure and without any precursors for gelation. Furthermore, the system can provide a sustained release of the model drug BSA at a therapeutic concentration for at least two months. The biphasic release mechanism of the system can block the initial higher amount of VEGF during burst-release state and keep the drug concentration at therapeutic windows while in its second steady-release state, preventing retinal tissues from toxic drug concentration. Another advantage of the proposed system is that the use of nanosized particles instead of microsized ones allows the system to provide a higher bioavailability of the drugs, which is achieved by penetration of the nanoparticles into the RPE layers thus extending the drug’s retention time in the posterior segment of the eye. However, an optimal preparation method and the activity and stability of the released protein should be confirmed in a future study.

In conclusion, the proposed composite DDS was easily prepared with biocompatible materials and it is promising for prolonged and sustained release of model protein drugs to the posterior segment of the eyes, which can reach better therapeutic effect for a chronic neovascular ocular disease like AMD. For future research, with bevacizumab and elevating the in vivo efficacy of the system is necessary to determine overall effectiveness for clinical use.

## Figures and Tables

**Table 1:** Abbreviations of the tested groups and their compositions.

Group	HA-VS ^1^ (% *w*/*v*)	HA-SH ^2^ (% *w*/*v*)	BSA ^3^ (mg)	BSA-laden NP ^4^ (mg)	Total Polymer Concentration (% *w*/*v*)
A ^5^	1	2	0	0	1.5
A-BSA ^6^	1	2	1	0	1.5
A-NP ^7^	1	2	0	20	1.5
B ^8^	2	4	0	0	3
B-BSA ^9^	2	4	1	0	3
B-NP ^10^	2	4	0	20	3
NP ^11^	0	0	0	20	0

^1^ Divinyl sulfone functionalized hyaluronan polymer. ^2^ Thiolated hyaluronan polymer. ^3^ Bovine serum albumin. ^4^ Bovine serum albumin loaded PLGA nanoparticle. ^5^ Hydrogel containing 1% *w*/*v* HA-VS polymer and 2% *w*/*v* HA-SH polymer. ^6^ Hydrogel containing 1% *w*/*v* HA-VS polymer, 2% *w*/*v* HA-SH polymer, and 1 mg of BSA. ^7^ Composite DDS containing 1% *w*/*v* HA-VS polymer, 2% *w*/*v* HA-SH polymer, and 20 mg of BSA-laden NP. ^8^ Hydrogel containing 2% *w*/*v* HA-VS polymer and 4% *w*/*v* HA-SH polymer. ^9^ Hydrogel containing 2% *w*/*v* HA-VS polymer, 4% *w*/*v* HA-SH polymer, and 1 mg of BSA. ^10^ Composite DDS containing 2% *w*/*v* HA-VS polymer, 4% *w*/*v* HA-SH polymer, and 20 mg of BSA-laden NP. ^11^ Group containing 20 mg of BSA-laden NP only.

**Table 2:** Abbreviation and composition of the tested groups for particle behavior study.

Group	HA-VS (% *w*/*v*)	HA-SH (% *w*/*v*)	PS ^1^ Microparticle (µL)	PS Nanoparticle (µL)	Total Polymer Concentration (% *w*/*v*)
A-NP-50nm ^2^	1	2	0	1.25	1.5
A-NP-2µm ^3^	1	2	1.25	0	1.5
B-NP-50nm ^4^	2	4	0	1.25	3
B-NP-2µm ^5^	2	4	1.25	0	3

^1^ Polystyrene. ^2^ Composite DDS containing 1% *w*/*v* HA-VS polymer, 2% *w*/*v* HA-SH polymer, and 1.25 µL of PS nanoparticle. ^3^ Composite DDS containing 1% *w*/*v* HA-VS polymer, 2% *w*/*v* HA-SH polymer, and 1.25 µL of PS microparticle. ^4^ Composite DDS containing 2% *w*/*v* HA-VS polymer, 4% *w*/*v* HA-SH polymer, and 1.25 µL of PS nanoparticle. ^5^ Composite DDS containing 2% *w*/*v* HA-VS polymer, 4% *w*/*v* HA-SH polymer, and 1.25 µL of PS microparticle.

## Data Availability

The data presented in this study are available on request from the corresponding author.
